# Identification of Putative Molecules for Adiponectin and Adiponectin Receptor and Their Roles in Learning and Memory in *Lymnaea stagnalis*

**DOI:** 10.3390/biology12030375

**Published:** 2023-02-27

**Authors:** Kanta Fujimoto, Yuki Totani, Junko Nakai, Nozomi Chikamoto, Kengo Namiki, Dai Hatakeyama, Etsuro Ito

**Affiliations:** 1 Department of Biology, Waseda University, Tokyo 162-8480, Japan; 2 Faculty of Pharmaceutical Sciences, Tokushima Bunri University, Tokushima 770-8514, Japan; 3 Graduate Institute of Medicine, School of Medicine, Kaohsiung Medical University, Kaohsiung 80708, Taiwan

**Keywords:** adiponectin, adiponectin receptor, C1q family, escape behavior, hemolymph glucose concentration, insulin, *Lymnaea*, molluscan insulin-related peptide, operant conditioning

## Abstract

**Simple Summary:**

Insulin and insulin-like peptides are involved in improving learning and memory in both vertebrates and invertebrates. Adiponectin has blood glucose-lowering and insulin sensitivity-increasing effects in mammals. These two facts led us to hypothesize that adiponectin and its receptors play an important role in learning and memory. We evaluated this hypothesis using the pond snail *Lymnaea stagnalis*, which has long been used for studies of learning and memory. First, the genes coding for putative molecules of adiponectin and its receptor in *Lymnaea* were identified, and then their localization in the central nervous system and changes in their expression levels associated with the nutritional conditions were examined. Next, an operant conditioning protocol of escape behavior was applied to the snails, and changes in the expression levels of adiponectin and its receptor were examined. Adiponectin was upregulated by food deprivation, whereas the expression of its receptor was upregulated after operant conditioning was established. These findings suggested the involvement of the adiponectin-signaling cascade in learning and memory in *Lymnaea* via changes in the concentrations of glucose and the activation of insulin.

**Abstract:**

Adiponectin enhances insulin sensitivity, which improves cognition in mammals. How adiponectin affects the mechanism’s underlying cognition, however, remains unknown. We hypothesized that experiments using the pond snail *Lymnaea stagnalis*, which has long been used in learning and memory studies and in which the function of insulin-like peptides affect learning and memory, could clarify the basic mechanisms by which adiponectin affects cognition. We first identified putative molecules of adiponectin and its receptor in *Lymnaea*. We then examined their distribution in the central nervous system and changes in their expression levels when hemolymph glucose concentrations were intentionally decreased by food deprivation. We also applied an operant conditioning protocol of escape behavior to *Lymnaea* and examined how the expression levels of adiponectin and its receptor changed after the conditioned behavior was established. The results demonstrate that adiponectin and adiponectin’s receptor expression levels were increased in association with a reduced concentration of hemolymph glucose and that expression levels of both adiponectin and insulin-like peptide receptors were increased after the conditioning behavior was established. Thus, the involvement of the adiponectin-signaling cascade in learning and memory in *Lymnaea* was suggested to occur via changes in the glucose concentrations and the activation of insulin.

## 1. Introduction

The pond snail *Lymnaea stagnalis* is an important model system for studying the causal neuronal mechanisms of associative learning and the subsequent formation of long-term memory. *Lymnaea* can be both classically and operantly conditioned for a number of different behaviors, and researchers have primarily focused on feeding, withdrawal, and aerial respiratory behaviors [1,2,3,4,5]. *Lymnaea* possesses relatively simple nervous systems, and the neuronal circuits mediating many of the behaviors that exhibit learning and memory have been well elucidated. Many of the identified neurons in the circuits mediating the behaviors are large, and they can consistently be recorded from individuals that have been subjected to either learning or control procedures [6].

Insulin-like peptides in the brain are strongly involved in the learning and memory mechanisms of *Lymnaea* [7,8,9,10]. For example, in long-term memory formation following a conditioned taste aversion (CTA) protocol [9], molluscan insulin-related peptides (MIPs) are up-regulated at the mRNA level in the *Lymnaea* central nervous system (CNS) [11], and MIPs enhance the neural transmission efficacy at a synapse in the CTA neural circuit [12]. A common role of insulin and insulin-like peptides is to lower blood glucose levels, and MIPs lower hemolymph glucose levels in *Lymnaea* [13]. CTA learning performance in *Lymnaea* is best during mild starvation (i.e., food deprivation for 1 day), but learning and memory deteriorate during severe starvation (i.e., food deprivation for 5 days) [13]. In addition, injections of mammalian insulin into *Lymnaea* improve learning and memory performance in 5-day food-deprived snails [13]. The relationships between the multiple functions of MIPs, nutritional status, and learning ability have been investigated [13].

Adiponectin, a ca. 30-kDa adipokine secreted by adipocytes, has blood glucose-lowering and insulin resistance-improving effects in mammals [14,15]. Thus, adiponectin is often discussed in connection with type 2 diabetes mellitus and metabolic syndrome [16,17,18]. Adiponectin belongs to the C1q family, with a collagen domain on the N-terminal and a globular C1q domain on the C-terminal [19]. In mammals, AdipoR1, AdipoR2, and T-cadherin are reported as adiponectin receptors [20,21]. AdipoR1 and AdipoR2, in particular, whose structures are 7-transmembrane receptors, are thought to be involved in the insulin resistance-improving effects of adiponectin [16]. Unlike the widely known 7-transmembrane G protein-coupled receptor (GPCR), however, the N-terminal is intracellular and the C-terminal is extracellular [22]. Adiponectin receptor-like proteins have also been studied in molluscs, and such proteins have been identified in the Japanese oyster *Crassostrea gigas* and are implicated in immune responses [23].

The function between the C1q protein and adiponectin is different in mammals: they complement system and glucose-level regulation, respectively. In molluscs, several C1q-related and C1q-domain-containing proteins were isolated, and these proteins are suggested to be involved in molluscan immune systems [24,25,26]. However, in mammals, adiponectin can bind with the C1q complex and activate the classical complement pathway [27]. Additionally, adiponectin is structurally homologous to C1q and can stimulate the tyrosine kinase-dependent engulfment of apoptotic cells through a shared pathway [28]. Taken together, we speculate that the *Lymnaea* C1qC, which will be identified in the present study, harbors a similar function to adiponectin.

Adiponectin also has a role in the brain and its function has been studied in relation to “dementia”. For example, in rat models of dementia, adiponectin improves memory and neuroplasticity [29,30]. Mice with AdipoR1 knockdown or chronic adiponectin deficiency exhibit impaired insulin signaling and impaired learning and memory [31,32]. Caloric restriction leads to increased serum insulin and adiponectin concentrations, and improved insulin sensitivity and hippocampus-dependent spatial learning ability in mice [33]. Although the reports mentioned above suggest a relationship between adiponectin function and learning and memory, the detailed mechanisms of this relationship remain unclear. Therefore, we investigated the further possible involvement of adiponectin in learning and memory using *Lymnaea stagnalis*, which has long been used for neuroscience studies. First, we identified the genes coding putative molecules corresponding to adiponectin (LymAdipo) and *Lymnaea* adiponectin receptor (LymAdipoR) in *Lymnaea*, and then examined their localization in the CNS and changes in their expression levels associated with the nutritional status and the formation of operant conditioning of escape behavior. Benatti et al. recently revisited the operant conditioning of escape behavior that we had previously studied [34], and it was again attracting attention [35]. We thus wanted to broaden the scope of our research into the molecular mechanisms of operant conditioning as well as classical conditioning.

## 2. Materials and Methods

### 2.1. Snails

*Lymnaea stagnalis* with a 20 to 25 mm shell length obtained from our snail-rearing facility (original stocks from Vrije Universiteit Amsterdam) were used. They were fed turnip leaves (*Brassica rapa* var. *perviridis*, known as Komatsuna in Japanese) ad libitum and kept in dechlorinated tap water under a 12 h light:12 h dark cycle at 21.0–22.5 °C.

### 2.2. Definition of Nutritional Status

The nutritional status of the snails was defined as follows [36]: (1) “Day 0” = snails food-deprived; (2) “Day −1” = snails fed normally; (3) “Day 1” = snails food-deprived for 1 day; (4) “Day 5” = snails food-deprived for 5 days; and (5) “High Glucose” (HG) = snails immersed in 20 mM sucrose instead of turnip leaves for 2 days.

### 2.3. Identification of LymAdipo and LymAdipoR

Putative molecule sequences for adiponectin and its receptor in *Lymnaea* (LymAdipo and LymAdipoR) were identified by a BLAST search (https://blast.ncbi.nlm.nih.gov/Blast.cgi (accessed on 26 February 2023)) using the transcriptome shotgun assembly (TSA) database for *Lymnaea* [37] on the basis of the adiponectin receptors in the Japanese oyster *Crassostrea gigas* (XM_011441672.3). The domain sequences of the identified amino acid sequences were predicted with the database Pfam in InterPro (http://www.ebi.ac.uk/interpro/ (accessed on 26 February 2023)) and another database, the Simple Modular Architecture Research Tool (SMART) (http://smart.embl-heidelberg.de (accessed on 26 February 2023)). Furthermore, the transmembrane regions were predicted with the database Phobius in InterPro. Multiple alignments were analyzed using ClustalW (https://www.genome.jp/tools-bin/clustalw (accessed on 26 February 2023)). The maximum likelihood trees of adiponectin-like and adiponectin receptor-like proteins identified from different organisms were generated using MEGA 11 software (https://www.megasoftware.net/ (accessed on 26 February 2023)). The amino acid sequences used for the phylogenic tree are listed in Table 1.

### 2.4. In Situ Hybridization

In situ hybridization was performed according to a previous study [38] with modifications. For hybridization, frozen sections were then prepared as follows: the CNS was isolated from each anesthetized *Lymnaea* in chilled saline (NaCl 50 mM, KCl 1.6 mM, MgCl_2_ 2 mM, CaCl_2_ 3.5 mM, HEPES 10 mM, pH 7.9), fixed in 4% paraformaldehyde in phosphate-buffered saline (PBS) for 1 h at room temperature, and then washed with PBS containing 30% sucrose. After embedding the fixed CNS in an OCT compound (Sakura Finetek, Tokyo, Japan), serial 10 µm-thick frozen sections were cut horizontally on a cryostat (CM3000; Leica, Nussloch, Germany) and placed on MAS-coated glass slides (Matsunami glass, Osaka, Japan).

For probe synthesis, regions of probes for LymAdipo and LymAdipoR were amplified from *Lymnaea* cDNA with Ex Taq (Takara Bio, Shiga, Japan), and the primers are listed in Table 2. The polymerase chain reaction (PCR) products were cloned into pTAC-2 plasmids. The inserted regions were amplified with KOD FX (Toyobo, Osaka, Japan) and forward and reverse M13 primers, and then purified using NucleoSpin Gel and PCR Clean-up (Takara). The sense and antisense probes were synthesized at 37 °C for 2 h with MAXIscrip SP6/T7 Transcription Kit (Invitrogen-Thermo Fisher Scientific, Waltham, MA, USA) and RNA labeling mix (DIG-UTP; Roche, Basel, Switzerland).

Details of the in situ hybridization experiments were the same as those in Hatakeyama’s study [38]. The slides with CNS sections were overlaid with Immu-Mount (Shandon, Fisher Scientific, Singapore), and cover-slipped for light microscopic examination (CKX53; Olympus, Tokyo, Japan).

### 2.5. Real-Time PCR

The real-time PCR protocol was performed according to the previous study [36] with modification. The snail’s CNS was dissected and stored at −80 °C. In the experiments, to identify the ganglion expressing the target mRNAs, the samples were separated by ganglion type, and three individual ganglia were collected together as a single sample, whereas the whole CNS was collected in the other experiments. Total RNA was extracted using ISOGEN II (311-07361; Nippon Gene, Tokyo, Japan) according to the manufacturer’s instructions. cDNA was synthesized by the ReverTra Ace qPCR RT Master Mix with gDNA Remover (Toyobo). THUNDERBIRD Next SYBR qPCR Mix (Toyobo) was used to perform real-time PCR (StepOnePlus Real-Time PCR System; Applied Biosystems, Waltham, MA, USA). The relative mRNA levels were quantified using the comparative Ct method. The Ct values of the target genes were normalized by dividing by the mean of the Ct values of 18S ribosomal RNA and β-tubulin. The mean of 18S ribosomal RNA and β-tubulin exhibited stable values under the measured conditions. The primer sequences are shown in Table 2. Efficiency values for the real-time PCR primers ranged from 90 to 110%. The PCR conditions were as follows: 1 cycle at 95 °C for 30 s, followed by 40 cycles of denaturation at 95 °C for 5 s; and annealing at 60 °C for 10 s. Melting curve analysis was performed from 60 to 95 °C with a heating rate of 0.3 °C/s.

### 2.6. Measurement of the Hemolymph Glucose Concentration

Moisture around the *Lymnaea* abdomen was thoroughly wiped off with a paper towel, and the tip of a pipette was used to stimulate the mantle. Upon stimulation, 150 µL of hemolymph was collected from the snail’s renal hole. The glucose concentration in the collected hemolymph was measured using a Glucose Assay Kit-WST (Dojindo, Kumamoto, Japan). The working solution for 10 experimental wells was prepared from Dye Mixture Stock Solution:Assay Buffer:Enzyme Stock Solution, which were contained in the kit, at a ratio of 50 µL:450 µL:9 µL. In each well, 50 µL of hemolymph and 50 µL of the working solution were added. After incubation at 37 °C for 30 min, absorbance at 450 nm was measured using a microplate reader (Corona Electronic, Ibaraki, Japan). For calibration, a serial dilution series of 0.5, 0.25, 0.125, 0.0625, 0.0313, 0.0157, 0.00785, and 0 mmol/L of glucose standard was used. The HG snails were compelled to swim in the rearing water (i.e., tap water) for 30 min prior to the collection of hemolymphs to exclude glucose solution on the body surface.

### 2.7. Operant Conditioning of Escape Behavior

The operant conditioning protocol for the escape behavior experiments was constructed based on previous studies [34,35]. A tray was lined with paper towels soaked with distilled water (DW, neutral reinforcement) or 100 mM KCl (negative reinforcement), and 35 mm dish lids were placed on the tray. These lids were filled with DW at a depth of 3 mm, and a *Lymnaea* was placed in the center of each lid. The experiments comprised three steps: the first step was an 80 min pre-test. Both the negative and neutral reinforcement groups were placed on paper towels soaked with DW. The second step was a 60 min training period. The negative reinforcement group was placed on paper towels soaked with 100 mM KCl, and the neutral reinforcement group was placed on paper towels soaked with DW. The third step was a 60 min post-test. Both groups were placed on paper towels soaked with DW. In all three tests, the number of escapes from the lid was recorded every 20 min. The timing of the escape was defined as the moment when the *Lymnaea* leaned out of the lid and put its head on the paper towel. When the escape was confirmed, the snail was relocated to the center of the lid. Besides recording the number of escapes per 20 min, the latency to the first escape was recorded in the pre-test and post-test. For individuals that did not escape within 60 min, the first escape was considered to have a 60 min latency. The CNS was dissected from *Lymnaea* 60 min after the post-test and subjected to real-time PCR experiments.

### 2.8. Statistics

The data are expressed as mean ± standard error of the mean (SEM). N indicates the biological replications unless otherwise noted. Significant differences (*p* < 0.05) between two groups were determined by Student’s *t*-test or Welch’s *t*-test. Comparisons of three or more groups were made by one-way ANOVA. Tukey‘s test was used for multiple comparisons when significant differences were observed and the homogeneity of variances could be assumed. This statistical calculation was applied to the data of real-time PCR for LymAdipoR for the ganglia and the data of mRNA expression level changes of Adipo, AdipoR, and MIPR under different nutritional conditions. Games–Howell‘s test was used when significant differences were observed but the homogeneity of variances could not be assumed. This was applied to the data of real-time PCR for LymAdipo for the ganglia and the data of mRNA expression level changes of the MIP II data under different nutritional conditions. Comparisons in changes in escape behavior were made by two-way repeated measures ANOVA. In this case, multiple comparisons were performed using the Bonferroni test. SPSS Statistics 28 was used for the statistics.

## 3. Results

### 3.1. Identification of Putative Molecules of Adiponectin and Its Receptor in Lymnaea

**Figure 1:**
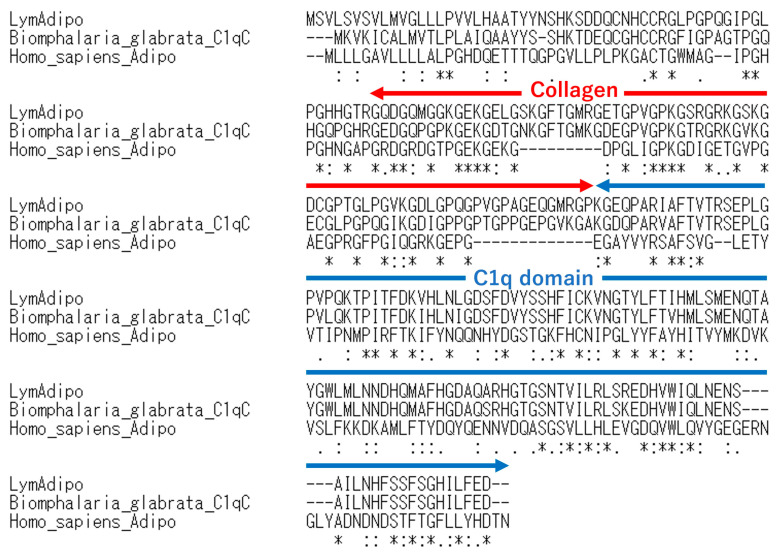
Comparison of deduced amino acids of LymAdipo with others. LymAdipo: *Lymnaea stagnalis* adiponectin; Biomphalaria_glabrata_C1qC: C1qC of the freshwater snail *Biomphalaria glabrata*; Homo_sapiens_Adipo: human (*Homo sapiens*) adiponectin. Collagen indicates collagen-like repeat sequences. * indicates the conserved amino acid and the colon (:) and period (.) indicate high or weak similarity of the properties of the amino acids, respectively.

A putative adiponectin in *Lymnaea*, LymAdipo, was identified from the homolog of the freshwater snail *Biomphalaria glabrata* C1qC (XM_013227908.1). The Blastn (Standard Nucleotide BLAST) search resulted in hits for *Lymnaea stagnalis* mRNA TSA (contig: LymstCNS_TSA_2863, mRNA sequence FX182981.1). Examination of this mRNA sequence revealed that it contained the full length of its open-reading frame (ORF). The sequence was translated for the ORF and aligned with adiponectin-like proteins from other organisms (Figure 1). The identified LymAdipo sequence has a collagen-like repeat sequence consisting of Gly-X-Y repeats as in human and mouse adiponectin, and a C1q domain, confirming that it belongs to the C1q family based on the Pfam database.

**Figure 2:**
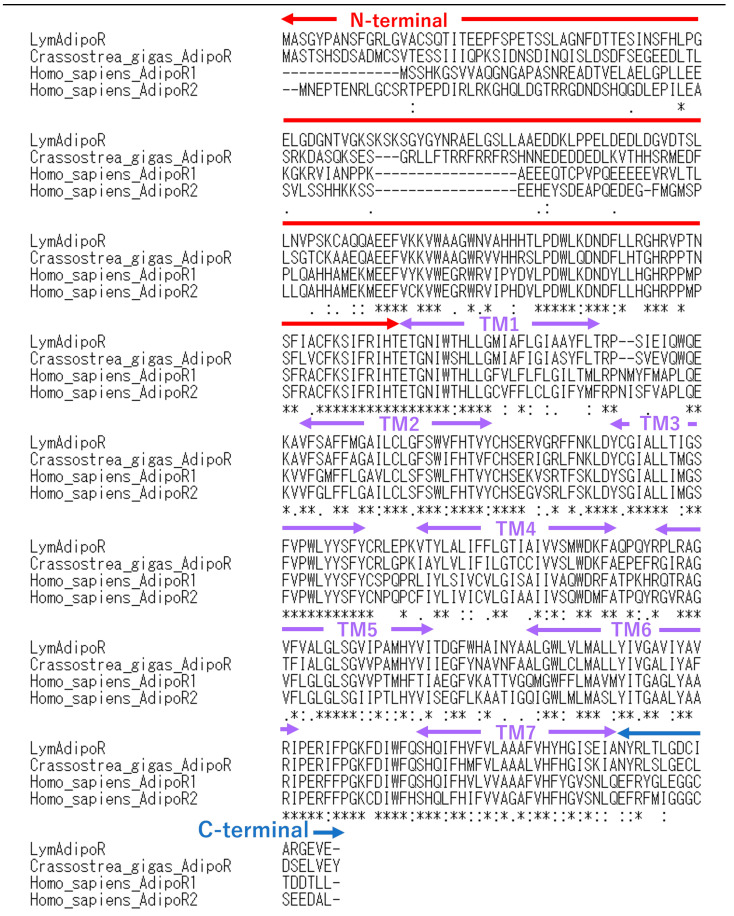
Comparison of deduced amino acids of LymAdipoR with others. LymAdipoR: *Lymnaea stagnalis* adiponectin receptor; Crassostrea_gigas_AdipoR: Japanese oyster (*Crassostrea gigas*) adiponectin receptor; Homo_sapiens_AdipoR1: human (*Homo sapiens*) adiponectin receptor type 1; Homo_sapiens_AdipoR2: human (*Homo sapiens*) adiponectin receptor type 2. * indicates the conserved amino acid. The colon (:) and period (.) indicate high and weak similarity of the properties of the amino acids, respectively. TM indicates the transmembrane regions numbered 1 through 7.

A putative adiponectin receptor in *Lymnaea*, LymAdipoR, was identified based on the Japanese oyster *Crassostrea gigas* adiponectin receptor (XM_011441672.3). The Blastn search resulted in hits for *Lymnaea stagnalis* mRNA TSA (contig: LymstCNS_TSA_1103, mRNA sequence FX181221.1). The sequence of this mRNA was examined and found to contain the full length of the ORF. Figure 2 shows the sequence translated from the ORF and the alignment with the adiponectin receptor-like proteins of other organisms. The predictions for the domain sequence and transmembrane region suggest that LymAdipoR has transmembrane regions and extracellular regions at the N- and C-terminals that are highly homologous with those of vertebrates. LymAdipoR comprises 404 amino acids and is well conserved between *Lymnaea* and mammals. LymAdipoR and human AdipoR1 or AdipoR2 displayed 47% or 50% amino acid similarity, respectively, and in particular, high amino acid similarity was observed in the transmembrane region.

**Figure 3:**
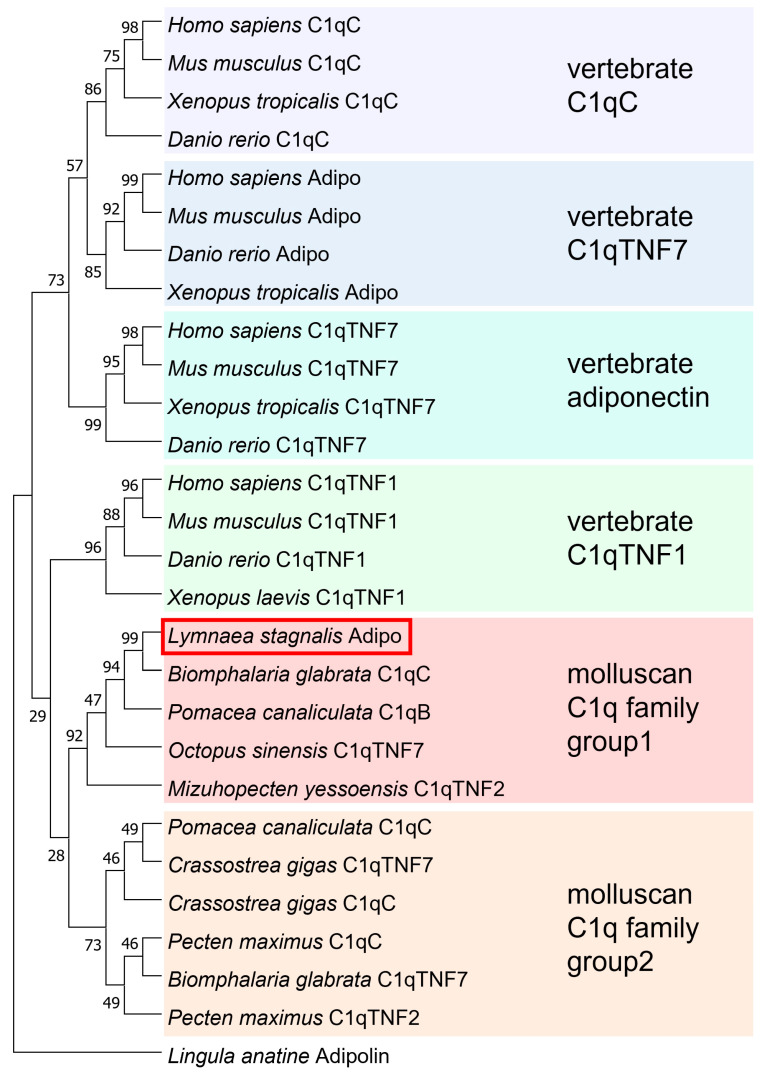
A molecular phylogenetic tree of 27 adiponectin-like proteins using the maximum likelihood method. The bootstrap value for each branch was calculated by testing the phylogenetic tree 1000 times and is expressed as a percentage.

**Figure 4:**
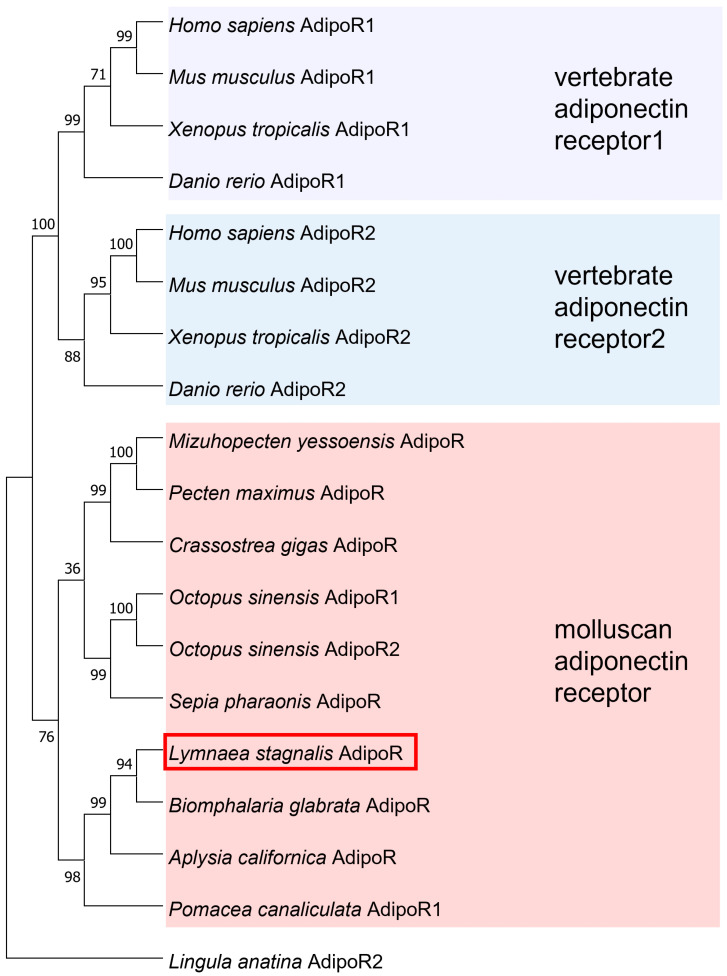
A molecular phylogenetic tree of 19 adiponectin receptor-like proteins using the maximum likelihood method. The bootstrap value for each branch was calculated by testing the phylogenetic tree 1000 times and expressed as a percentage.

A molecular phylogenetic tree of adiponectin-like proteins and their receptor-like proteins deduced from various animals was generated using the maximum likelihood method [39] (Figure 3 and Figure 4). The adiponectin-like sequence identified in the present study for *Lymnaea* (i.e., LymAdipo) is most similar to the C1qC of the freshwater snail *Biomphalaria glabrata*, and it was clustered into the C1q family of Cephalopoda and Gastropoda. This cluster was designated “molluscan adiponectin group 1”. C1q family molecules of bivalves such as the Japanese oyster *Crassostrea gigas* and the scallop *Pecten maximus* formed a separate cluster, which was designated “molluscan adiponectin group 2”. In contrast to the strict clustering of C1q family members in vertebrates (for example, the C1qC cluster is different from the adiponectin cluster), various types of C1q family molecules are intermingled within a single cluster in molluscs, suggesting that the subdivision of C1q family molecules is underdeveloped in invertebrates. From these analyses, LymAdipo identified in the present study is considered to be the “primitive” adiponectin of molluscs.

LymAdipoR was first clustered with the adiponectin receptor-like protein of the freshwater snail *Biomphalaria glabrata* (Figure 4). It then formed a large cluster with adiponectin receptors of gastropods, cephalopods, and bivalves, and finally, it was clustered into the invertebrate adiponectin receptor branch. Due to the highly conserved transmembrane and N- and C-terminal extracellular regions, the sequence identified in the present study was used as LymAdipoR in the following experiments.

### 3.2. Localization of LymAdipo and LymAdipoR in the Lymnaea CNS

**Figure 5:**
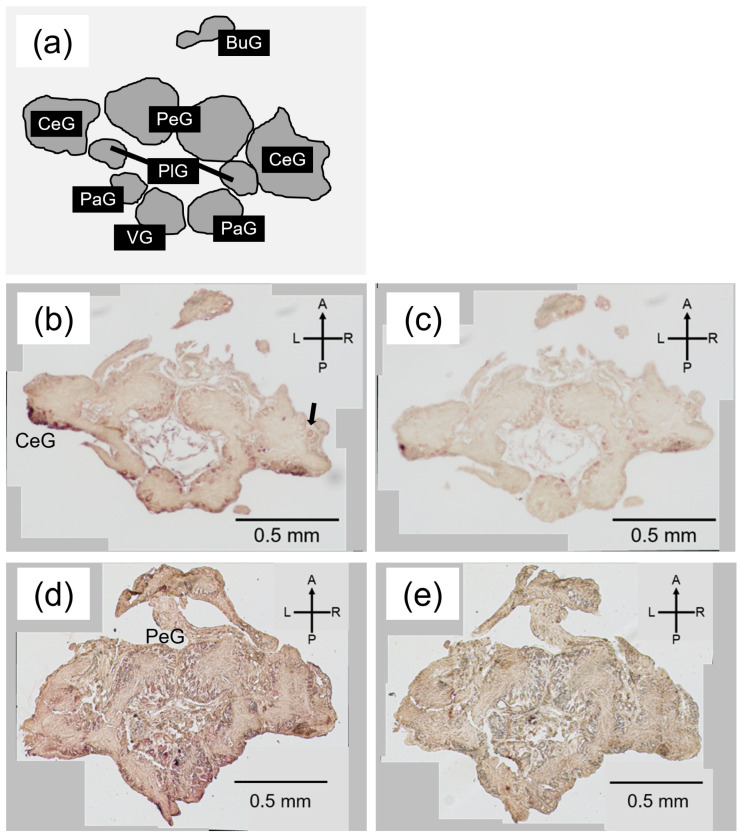
In situ hybridization of LymAdipo and LymAdipoR in *Lymnaea* CNS. (**a**) Notation of ganglia. BuG: buccal ganglion, PeG: pedal ganglion, CeG: cerebral ganglion, PlG: pleural ganglion, PaG: parietal ganglion, VG, visceral ganglion. (**b**) Staining of the antisense probe for LymAdipo. Arrow points to the cerebral giant cell. (**c**) Staining of the sense probe for LymAdipo. (**d**) Staining of the antisense probe for LymAdipoR. (**e**) Staining of the sense probe for LymAdipoR. CeG: cerebral ganglion, PeG: pedal ganglion. Scale bars: 0.5 mm.

**Figure 6:**
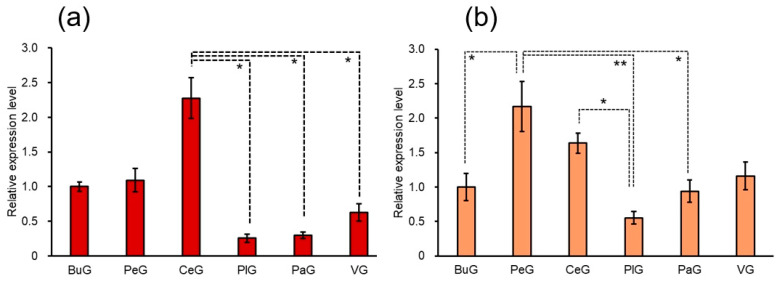
Real-time PCR for LymAdipo (**a**) and LymAdipoR (**b**) in *Lymnaea* CNS. The mRNA expression levels (RQ values) are expressed in relative expression as the data of the buccal ganglia are set as 1 for easy comparison. * *p* < 0.05, ** *p* < 0.01. N = 5 each.

To identify the CNS localization of LymAdipo and LymAdipoR, we performed in situ hybridization using frozen sections of the whole CNS and real-time PCR quantitation for mRNA extracted from each ganglion (Figure 5 and Figure 6).

In situ hybridization with the antisense probe showed the presence of LymAdipo signals mainly in the cerebral ganglia, parietal ganglia, and visceral ganglion (Figure 5b), whereas these signals were not observed with the sense probe (Figure 5c). The signals were recognized in the cerebral giant cells, which are regulatory neurons and play important roles in various behaviors [40]. The hybridization signals for the LymAdipoR antisense probe were observed mainly in the pedal ganglia, parietal ganglia, and visceral ganglion (Figure 5d). As with LymAdipo, no signals were observed with the sense probe for LymAdipoR (Figure 5e).

The results of real-time PCR quantification for mRNA extracted from each ganglion are shown in Figure 6. The real-time PCR results show the expression of LymAdipo in all the ganglia. In particular, the expression level of LymAdipo in the cerebral ganglia was significantly higher than that in the other ganglia (*F*(5,24) = 19.001, *p* < 0.001, cerebral ganglia vs. plural ganglia: *p* = 0.018; cerebral ganglia vs. parietal ganglia, *p* = 0.020; cerebral ganglia vs. visceral ganglion: *p* = 0.032, N = 5 each). LymAdipoR was also expressed in all the ganglia. The highest expression was found in the pedal ganglia, followed by the cerebral ganglia (*F*(5,24) = 5.902, *p* = 0.004, pedal ganglia vs. buccal ganglia: *p* = 0.020; pedal ganglia vs. plural ganglia: *p* < 0.001, pedal ganglia vs. parietal ganglia: *p* = 0.013: cerebral ganglia vs. plural ganglia: *p* = 0.035, N = 5 each).

### 3.3. Glucose Concentrations in the Hemolymph and Changes in the LymAdopo and LymAdipoR mRNA Expression Levels under Different Nutritional Conditions

The hemolymph glucose concentrations under the three food-deprivation conditions (Day −1, Day 1, and Day 5) and HG state were quantified with a glucose concentration kit. The glucose concentrations were (mean ± SEM, mmol/L): 0.104 ± 0.017 in HG snails, 0.043 ± 0.005 in Day −1 snails, 0.035 ± 0.006 in Day 1 snails, 0.022 ± 0.003 in Day 5 snails (N = 13 each). The glucose concentration decreased as food deprivation progressed (*p* = 0.0145 between Day −1 and Day 5). When *Lymnaea* were reared in a 20-mM sucrose solution for 2 days (referred to as HG), the glucose concentrations in the hemolymph of the HG cohort were significantly higher than those in the hemolymph of the Day −1 snails (*p* = 0.0047, N = 13 each), demonstrating that the HG snails were hyperglycemic individuals.

**Figure 7:**
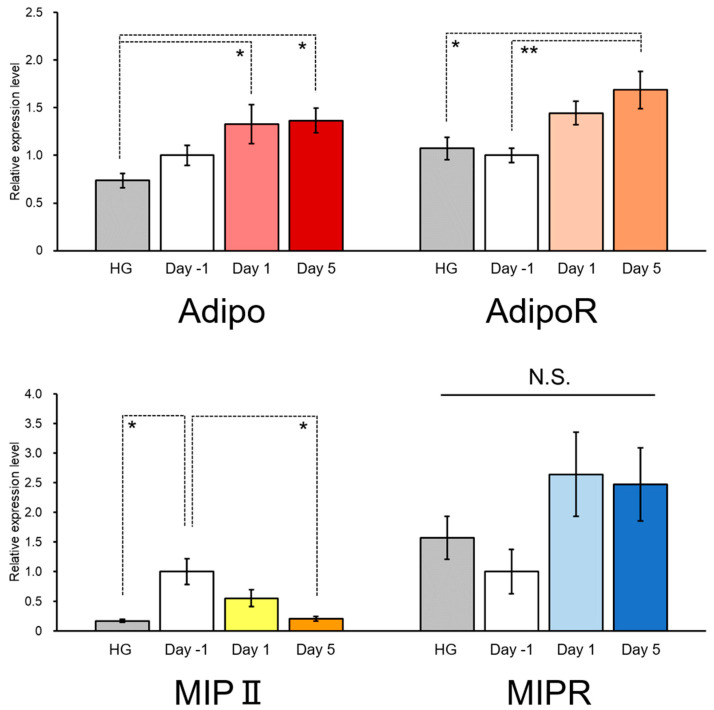
Changes in LymAdipo, LymAdipoR, MIP II, and MIPR mRNA expression levels in the whole CNS under different nutritional conditions. The expression levels of each gene are expressed as relative expression level (RQ) as the level in Day −1 is set as 1. * *p* < 0.05, ** *p* < 0.01. N = 10 each.

Due to the fact that the glucose concentration in the hemolymph changes is associated with changes in the nutritional conditions, we expected that the mRNA expression level of LymAdipo may also change. We thus measured the expression levels of LymAdipo and LymAdipoR in the whole CNS by real-time PCR (Figure 7). The results show that the expression levels of both LymAdipo and LymAdipoR were significantly higher than those of HG as food-deprivation progressed (LymAdipo. *F*(3,36) = 4.308, *p* = 0.011, HG vs. Day 1: *p* = 0.029; HG vs. Day 5: *p* = 0.018. LymAdipoR. *F*(3,36) = 5.163, *p* = 0.005, HG and Day 5: *p* = 0.021; Day −1 vs. Day 5: *p* = 0.008, N = 10 each).

The mRNA levels of molluscan insulin-related peptide II (MIP II) were also examined (Figure 7). Contrary to expectations, the expression of MIP II mRNA was significantly lower in the hyperglycemic HG snails than in the normally fed Day −1 snails (*F*(3,36) = 7.721, *p* = 0.010, HG and Day −1: *p* = 0.023, N = 10 each), and it decreased with the initiation of food deprivation, becoming significantly lower in Day 5 snails than in Day −1 snails (Day −1 and Day 5: *p* = 0.032). MIP II expression was as low in Day 5 snails as in HG snails in a hyperglycemic condition. The expression levels of the only receptor for MIPs, the MIP receptor (MIPR), were examined, and no significant differences among the four nutritional states were detected (*F*(3,36) = 1.861, *p* = 0.154, N = 10 each).

### 3.4. Establishment of Escape Behavior by Operant Conditioning and Change in the Expression Levels of LymAdipo and LymAdipoR during Memory Formation

**Figure 8:**
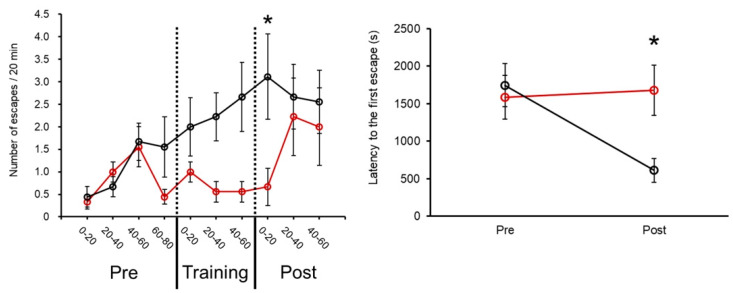
Changes in escape behavior before and after operant conditioning using the negative (KCl) and neutral (DW) reinforcements. Left panel. Number of escape attempts per 20 min. *y*-axis shows the number of escape attempts in 20 min, and the *x*-axis shows time. Right panel. Latency in escape behavior in pre- and post-tests. If the latency was over 3600 s, such individuals were judged to have an escape time of 60 min. The red line indicates the negative reinforcement cohort, and the black line indicates the neutral cohort. * *p* < 0.05. N = 9 each.

The behavioral changes in escape behavior by operant conditioning were examined (Figure 8). The negative reinforcement cohort (i.e., KCl stimulation) had significantly fewer escape attempts than the neutral reinforcement cohort (i.e., DW stimulation) in the first 20 min of the posttest period (*F*(9,144) = 2.170, *p* = 0.027 for interactions, *p* = 0.025 by Bonferroni test, N = 9 each), whereas there was no significant difference in the first 20 min of the pre-test period (*p* = 0.148 by Bonferroni test, N = 9 each) (Figure 8a).

The latency between the first escape attempt in the pre- and post-tests showed that the latency of the negative reinforcement cohort did not change between the pre- and post-tests (*F*(1,16) = 9.280, *p* = 0.008 for interactions, *p* = 0.742 by Bonferroni test, N = 9 each), whereas it decreased in the posttest of the neutral reinforcement cohort (*p* = 0.001 by Bonferroni test, N = 9 each) (Figure 8b). The latency did not differ significantly between the negative reinforcement cohort and the neutral reinforcement cohort in the pretests (*p* = 0.711 by Bonferroni test, N = 9 each), whereas there was a significant difference between them in the posttests (*p* = 0.015 by Bonferroni test, N = 9 each). Based on these results, we judged that an operant conditioning of escape behavior using an aversive stimulus was established.

**Figure 9:**
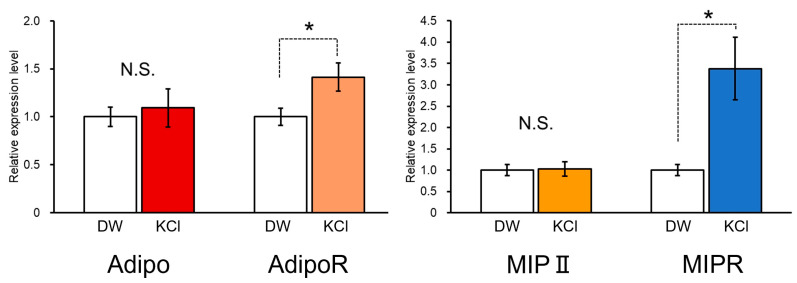
Real-time PCR for LymAdipo, LymAdipoR, MIP II, and MIPR mRNA after memory formation following operant conditioning of escape behavior. The mRNA expression levels (RQ values) are expressed as relative expression because the data of the neutral reinforcement are set as 1 for easy comparison. * *p* < 0.05. N = 9 each.

Changes in the mRNA expression levels of LymAdipo, LymAdipoR, MIP II, and MIP R associated with the operant conditioning of escape behavior were examined by real-time PCR (Figure 9). The results show that the expression of LymAdipoR and MIPR was significantly higher in the negatively reinforced (KCl) cohort than in the neutrally reinforced (DW) cohort (LymAdipoR: *p* = 0.0382, MIPR: *p* = 0.016, N = 9 each). The expression of LymAdipo was not different between the KCl cohort and the DW cohort. That of MIP II was also constant regardless of the negative or neutral reinforcement. These results suggest that the adiponectin-signaling cascade, especially including LymAdipoR, is involved in learning and memory in *Lymnaea* via the regulation of the hemolymph glucose concentration (Figure 7) and the activation of insulin signaling (Figure 9).

## 4. Discussion

We identified the putative molecules of adiponectin and its receptor in *Lymnaea*, LymAdipo, and LymAdipoR. LymAdipo, comprising 264 amino acids, has a Gly-X-Y collagen-like repeat at the N-terminal and a globular C1q domain at the C-terminal. These domains are common sequences in the C1q family of proteins, which includes adiponectin [19]. The results suggest that the C1q family proteins in molluscs are not as segmented as those in vertebrates and that they contain “primitive” adiponectin-like proteins.

As with mammalian adiponectin receptors, seven transmembrane regions were predicted in LymAdipoR, and the N-terminal intracellular region and the C-terminal extracellular region were revealed. The results suggest that LymAdipoR is a member of the progestin and adipoQ receptor (PAQR) family, to which the mammalian adiponectin receptor belongs, and that it possesses the sequences necessary for adiponectin receptor function. Vertebrates have two types of receptors, AdipoR1 and AdipoR2, whereas molluscs have only one type of receptor, with the exception of the octopus *Octopus sinensis*. That is, octopuses have two types of receptors. This also suggests that the subdivision of the adiponectin pathway occurred during post-vertebrate evolution and that molluscan adiponectin-like proteins are “primitive” [23].

The real-time PCR and in situ hybridization experiments revealed that LymAdipo is highly expressed in the cerebral ganglia, and LymAdipoR is highly expressed in the pedal ganglia and the cerebral ganglia. In the arcuate nucleus of the mouse hypothalamus, adiponectin and adiponectin receptors are co-expressed with leptin [41]. Leptin modifies feeding behavior by activating pro-opiomelanocortin (POMC)-expressing neurons [42], suggesting that adiponectin receptors in the mouse are involved in appetite suppression. In *Drosophila* larvae, adiponectin receptor-like proteins have been identified in neurons in the brain lobes, and are known to suppress the juvenile hormone (JH) response and promote insulin signaling [43]. In the present study, the main localization site of LymAdipo and LymAdipoR in the *Lymnaea* CNS was the cerebral ganglia. In particular, the cerebral giant cells in the cerebral ganglia are necessary for the control of feeding behavior including CTA in *Lymnaea* [40], and these contain LymAdipo and LymAdipoR. Taken together, we can speculate that the adiponectin-signaling cascade in *Lymnaea* may also function to regulate feeding behavior.

LymAdipoR expression was significantly increased by severe food deprivation (i.e., Day 5 snails). In the mouse brain, adiponectin receptors are upregulated during food deprivation [41], which is consistent with the findings in *Lymnaea*. However, because learning and memory performance in *Lymnaea* CTA is enhanced by mild food deprivation (i.e., Day 1 snails) [13,36], a positive effect of the adiponectin-signaling cascade on the learning and memory performance suggests that other factors (e.g., 5-HT concentration) that reduce the learning and memory performance are at work in Day 5 snails [36]. Alternatively, there may be an optimal LymAdipoR concentration range for adequate functioning of learning and memory capacity. The pedal ganglia, which most abundantly expressed LymAdipoR, regulates locomotion. The escape behavior is a kind of locomotion. AdipoR is also expressed in the cerebral ganglia, which is strongly involved in memory consolidation. On the other hand, HG individuals reared on a 20 mM sucrose for 2 days had significantly lower LymAdipo expression compared with severely food-deprived snails (Day 5). MIP II expression was significantly lower than that in Day –1 snails (i.e., the normal nutritional condition) and decreased to the same level as in Day 5 snails. These results suggest that the HG snails were in a “diabetic” state. The expression of adiponectin is reduced in obese rhesus macaques, which frequently develop type 2 diabetes mellitus [44].

On the other hand, we examined the voluntary movement to find whether a 20 mM sucrose solution affected *Lymnaea* behavior. The “active” or “quiescent” state, which was defined by Stephenson and Lewis [45], was studied using five snails each in the control and HG groups for 1 h. As a result, both groups were always in an “active” state and no difference was observed. The possibility that the HG sucrose solution causes osmotic stress to *Lymnaea* is unlikely because there is no change in their behavior. Furthermore, in our experiment, HG was only used to measure the expression level of genes, so whether it was osmotic stress or not was not an issue in this experiment. In the future, we believe that further caution will be necessary when performing behavioral experiments in the state of hyperglycemia.

MIP II expression decreased with progressive food deprivation. This is thought to be due to a decrease in hemolymph glucose levels, and thus a decrease in the need for insulin. It is also possible that when MIP II expression levels are low but sensitivity is high, the insulin spike becomes high, thereby establishing learning [13]. In the *Lymnaea* CNS, the expression of LymAdipoR increased and that of MIP II decreased with food deprivation, suggesting that the decreased expression of MIP II is compensated for by increasing the expression of LymAdipoR to increase insulin sensitivity.

The learning experiment used in the present study was operant conditioning of escape behavior, and thus the pedal ganglia and cerebral ganglia are important because escape can be learned by associating escape behavior with aversive stimuli. The high expression of LymAdipo and LymAdipoR in these characteristic ganglia leads us to imagine that the adiponectin-signaling cascade is somehow involved in escape behavior.

Regarding the fact that the latency did not decrease after training with KCl but decreased with DW, it is thought that this is because *Lymnaea* became accustomed to the dish lid environment through the experiment. In the KCl cohort, the habituation to the dish lid environment was hindered by the aversive stimulus to KCl, and as a result, there was no difference in latency between pre- and post-tests, and it is believed that the latency decreased only in the DW cohort.

We view the mechanism of operant conditioning of escape behavior as follows: Previous reports have already shown that the negative reinforcement did not weaken the *Lymnaea* mobility but made *Lymnaea* strictly understand to keep its position at a safe place [34]. The interaction between the neural pathways for withdrawal response and those for locomotion (foot-muscle extension) [46,47,48] is probably important. Therefore, in the future, we would like to deepen our understanding of the operant conditioning of escape behavior by performing experiments such as insulin injection in the same manner as for conditioned taste aversion [13].

## 5. Conclusions

The present study furthers our understanding that adiponectin-like proteins are possibly involved in the regulation of learning and memory. The research use of invertebrates, such as *Lymnaea*, is helpful to show the relationship among the actions of adiponectin, the function of insulin, and the regulation of learning and memory.

## Figures and Tables

**Table 1:** List of adiponectin-like and adiponectin receptor-like proteins and their accession numbers for maximum likelihood trees.

Protein	Accession Number
Homo sapiens Adipo	ABZ10942.1
Mus musculus Adipo	NP_033735.3
Xenopus tropicalis Adipo	XP_002938627.3
Danio rerio Adipo	NP_001373470.1
Biomphalaria glabrata C1qC	XP_013083362.1
Homo sapiens C1qC	AAH09016.1
Mus musculus C1qC	AAH43945.1
Xenopus tropicalis C1qC	XP_031750749.1
Danio rerio C1qC	AJP77512.1
Homo sapiens C1qTNF7	EAW92731.1
Mus musculus C1qTNF7	AAY21932.1
Xenopus tropicalis C1qTNF7	XP_017946491.1
Danio rerio C1qTNF7	XP_693031.2
Homo sapiens C1qTNF1	AAQ88790.1
Mus musculus C1qTNF1	AAY21926.1
Xenopus laevis C1qTNF1	XP_018094785.1
Danio rerio C1qTNF1	NP_001017875.1
Pomacea canaliculata C1qB	XP_025098395.1
Pomacea canaliculata C1qC	XP_025086549.1
Mizuhopecten yessoensis C1qTNF2	XP_021344498.1
Pecten maximus C1qC	XP_033753106.1
Pecten maximus C1qTNF2	XP_033744519.1
Octopus sinensis C1qTNF7	XP_029636373.1
Crassostrea gigas C1qC	XP_034310292.1
Crassostrea gigas C1qTNF7	XP_011449385.2
Biomphalaria glabrata C1qTNF7	KAI8775437.1
Lingula anatine Adipolin	XP_013384435.1
Homo sapiens AdipoR1	KAI4084514.1
Homo sapiens AdipoR2	KAI4064009.1
Mus musculus AdipoR1	AAH14875.1
Mus musculus AdipoR2	AAH24094.2
Crassostrea gigas AdipoR	XP_019926543.1
Danio rerio AdipoR1	NP_001314683.1
Danio rerio AdipoR2	NP_001020677.1
Xenopus tropicalis AdipoR1	NP_001007928.1
Xenopus tropicalis AdipoR2	XP_031754470.1
Drosophila melanogaster AdipoR	NP_651061.1
Octopus sinensis AdipoR1	XP_036366622.1
Octopus sinensis AdipoR2	XP_036366621.1
Aplysia californica AdipoR	XP_005097206.1
Pomacea canaliculata AdipoR1	XP_025108208.1
Mizuhopecten yessoensis AdipoR	XP_021370275.1
Lingula anatina AdipoR2	XP_013397290.1
Biomphalaria glabrata AdipoR	KAI8771842.1
Sepia pharaonis AdipoR	CAE1287042.1
Pecten maximus AdipoR	XP_033744837.1

**Table 2:** List of PCR primers.

Primer		Sequence (5′-3′)	Product Size (bp)
18S_PCR	Forward	CTC CTT CGT GCT AGG GAT TG	106
	Reverse	GTA CAA AGG GCA GGG ACG TA	
β-tubulin_PCR	Forward	CAA GCG CAT CTC TGA GCA GTT	108
	Reverse	TTG GAT TCC GCC TCT GTG AA	
LymAdipo_PCR	Forward	TGC TGA GCA TGG AGA ACC AG	111
	Reverse	CCG TGT TAC TTC CGG TTC CA	
LymAdipoR_PCR	Forward	TCC AGT GGC AAG AAA AGG CA	108
	Reverse	CAA CAC GTT CAC TGT GGC AG	
MIPII_PCR	Forward	AGA GGG CCA ATC ATC TTG CAG	77
	Reverse	GGA AGC CAG CCA AAT TCG AG	
MIPR_PCR	Forward	AGA CAG ACT ACT ATA GAA AAG GAG GTA AAG GAA	118
	Reverse	ACA ACT CCA TAT GAC CAA ACA TCT GA	
LymAdipo_in situ	Forward	TGC TGC CCG TAG TTC TAC AC	471
	Reverse	AGC TGT CTC CCA GGT TGA GA	
LymAdipoR_in situ	Forward	TTC TAT TGT CGC CTG GAG CC	421
	Reverse	AGT CCC CCA GGG TAA GTC TG	
M13	Forward	GTA AAA CGA CGG CCA GT	-
	Reverse	CAG GAA ACA GCT ATG AC	

## Data Availability

All data that support the findings of this study are available from the corresponding authors upon reasonable request.
